# Misalignment Fault Prediction of Wind Turbines Based on Improved Artificial Fish Swarm Algorithm

**DOI:** 10.3390/e23060692

**Published:** 2021-05-31

**Authors:** Zhe Hua, Yancai Xiao, Jiadong Cao

**Affiliations:** School of Mechanical, Electronic and Control Engineering, Beijing Jiaotong University, Beijing 100044, China; 17121266@bjtu.edu.cn (Z.H.); 19121256@bjtu.edu.cn (J.C.)

**Keywords:** misalignment, fault prediction, artificial fish swarm algorithm, least squares support vector machine

## Abstract

A misalignment fault is a kind of potential fault in double-fed wind turbines. The reasonable and effective fault prediction models are used to predict its development trend before serious faults occur, which can take measures to repair in advance and reduce human and material losses. In this paper, the Least Squares Support Vector Machine optimized by the Improved Artificial Fish Swarm Algorithm is used to predict the misalignment index of the experiment platform. The mixed features of time domain, frequency domain, and time-frequency domain indexes of vibration or stator current signals are the inputs of the Least Squares Support Vector Machine. The kurtosis of the same signals is the output of the model, and the 3σ principle of the normal distribution is adopted to set the warning line of misalignment fault. Compared with other optimization algorithms, the experimental results show that the proposed prediction model can predict the development trend of the misalignment index with the least prediction error.

## 1. Introduction

Energy shortage and environmental pollution have become two great challenges for human beings. Compared with oil and coal, wind energy has huge development potential as an environmentally friendly and renewable energy source. According to the report of the Global Wind Energy Council, the new installed capacity of global wind power in 2020 was 71.3 GW, which is 10.95 GW higher than that in 2019. Additionally, the cumulative installed capacity in 2020 was 721.86 GW. The wind energy industry is expected to achieve record growth in the next five years. It is estimated that more than 348 GW will be installed between 2020 and 2024. By the end of 2024, the total global installed capacity of wind power will be close to 1000 GW [1].

Wind turbines are generally installed in remote areas or sea areas with abundant wind resources, and their working environment is harsh and not convenient for timely maintenance. Therefore, faults often happen in wind turbines. The typical fault types are transmission system fault, blade fault, generator fault and gearbox fault. Misalignment is a kind of transmission system fault, which specifically occurs at the position where the output shaft of the gearbox and the rotor shaft of the generator are connected, and it is a slowly changing fault [2]. The symptoms before the fault are not obvious. As the running time increases, the degree of misalignment faults becomes more and more serious, which will affect the quality of power generation and cause the internal parts of the equipment to fail. Compared with sudden faults, slowly changing faults can use predictive algorithms to construct the mapping relationship between operating status and time, which can predict it in advance. Therefore, for the misalignment faults of wind turbines, reasonable and effective fault prediction technology should be used to predict the operation status of the equipment, and the fault information of future operation should notify the staff in advance to ensure the safe and stable operation of wind turbines.

Compared with the research on the diagnosis and prediction of the gearbox and bearing faults of the wind turbine, the research on the misalignment faults of the wind turbine is less. Reference [3] used the principle of “integrated learning” to combine various indicators into more reliable health indicators. The unsupervised algorithm was tested on a SCADA dataset covering two onshore wind farms with a total of 84 turbines operating for more than two years, and obtained 95.1% average accuracy. Reference [4] collected 50 real vibration data sets for analysis on a 2 MW wind turbine operating at the degradation of bearing performance, and the results showed that the regression model effectively improved the prediction performance of the artificial neural network. Reference [5] used the gearbox fault data obtained by the wind turbine monitoring and data acquisition system, using the confidence interval as the performance index, and proposed a new fault diagnosis and prediction method based on the support vector regression model. At present, some researchers have conducted research on misalignment faults. Reference [6] conducted multi-scale entropy research based on the angular and parallel misalignment of motor shaft, and applied back-propagation neural network to detect misalignment faults. Reference [7] was based on the vibration signal collected on a test bench simulating a wind turbine, and adopted support vector machine to effectively identify misalignment and imbalance faults. For the angle misalignment fault of the generator, Reference [8] proposed the track shape analysis to identify the angle misalignment fault type of the generator, and adopted a model-based method to identify the equivalent bending moment of the simulated shaft experimental vibration. It can be seen that most of the above research on misalignment faults is mainly focused on fault diagnosis, which can identify different types of misalignment faults. However, there are relatively few studies on the development trend of misalignment faults. This paper will select index that can characterize the development trend of misalignment faults, and make accurate predictions to realize early warning of faults.

In order to better predict the development trend of misalignment faults, the selection and construction of predictive models is very important. Commonly used prediction algorithms are Autoregressive Integrated Moving Average model (ARIMA), Random Forest (RF), Least Squares Support Vector Machine (LSSVM), Kalman Filter (KF), Long Short-Term Memory (LSTM), and so on. Table 1 lists the advantages and disadvantages of them.

According to the relatively complex structure of wind turbines and the influence of the external environment, the signals collected are often non-linear. Due to the limited faults samples collected in this paper, it can be seen from Table 1 that LSSVM can obtain higher prediction accuracy based on a small amount of sample data. Therefore, the LSSVM prediction algorithm is selected as the prediction model of misalignment faults. The principle of LSSVM is described in detail in reference [9]. Among them, the choice of penalty parameter and the radial basis function kernel in LSSVM has a great influence on its prediction accuracy. If the parameter is too large or too small, the generalization ability of the model will deteriorate, which will directly affect the prediction accuracy of the development trend of misalignment faults.

Considering the effectiveness and rationality of parameter optimization, this paper adopted an artificial intelligence optimization algorithm which is Artificial Fish Swarm Algorithm (AFSA) to optimize the parameters of the LSSVM prediction model. Some scholars have made improvements to AFSA and applied them to parameter optimization of prediction and classification algorithms. Reference [10] proposed an improved artificial fish swarm algorithm, which found the optimal parameters of the support vector regression by changing the behavior sequence and adapting the step length. The prediction accuracy of the heat transfer capacity was improved. Due to the shortcomings of the fixed step size of AFSA, Reference [11] adopted dynamic adjustments to the view and step size of the artificial fish. The simulation results showed that the convergence performance of the improved algorithm was significantly better than the original algorithm. For the shortcomings that AFSA is vulnerable to local minimums and the initial population is random, Reference [12] improved it by chaotic search. Experimental results showed that the improved algorithm had improved convergence and stability. According to the above references, the AFSA algorithm itself has certain shortcomings, such as random initialization parameters, the fixed searching vision and step, easily falling into the local optimal solution, which will affect the prediction accuracy of the development trend for misalignment faults.

Based on the above shortcomings of AFSA, an improved artificial fish swarm algorithm (IAFSA) is proposed, which combines chaotic maps with reverse Learning to produce a uniformly distributed initial population, adopts adaptive vision and step for parameter search, and introduce adaptive *t* distribution mutation to prevent premature maturity. This improved algorithm is used in the parameter optimization of the LSSVM to improve the accurate prediction of the development trend of misalignment faults. One of the contributions of this research is to explore the early fault symptoms in the time domain and frequency domain of the vibration and current signals when the misalignment fault occurs. The other main contribution of the research is to improve the AFSA to effectively search for the optimal solution of the LSSVM model, which can improve the prediction accuracy of the fault and reduce the false alarm rate.

## 2. Improvement of Artificial Fish Swarm Algorithm

Artificial Fish Swarm Algorithm was originally proposed by Xiaolei Li [13]. The algorithm imitates the life habit of fish swarm, and it has strong parallelism, robustness and global optimization ability [14]. In the optimization process of AFSA, the parameters to be optimized are the position of artificial fish, and the fitness value corresponding to each group of optimized parameters is the food concentration of artificial fish. In each iteration of the optimization process, each artificial fish will perform behaviors such as preying behavior, swarming behavior, and following behavior to update the position of the artificial fish until the location with the highest food concentration is found. Thus, the final optimal parameters are obtained.

However, there are still some problems in AFSA. For example, because AFSA initializes the parameters randomly, it cannot make the positions of the artificial fish evenly distributed in the solution space. Secondly, since the step and vision of AFSA are fixed, the search speed and optimization accuracy will be limited in the early and late iterations of the algorithm, which is easier to fall into the local optimal solution. In view of the above shortcomings, three improvements have been made based on AFSA.

### 2.1. Initialize the Population by Tent Maps and Reverse Learning

In order to make the positions of the artificial fish evenly distributed in the solution space, the chaotic maps method is adopted in the AFSA. Regularity, randomness, and ergodicity are characteristics of chaotic variables [15]. Common chaotic maps are logistic maps and tent maps. Research shows that tent maps have better ergodicity and uniformity than logistic maps [16]. Therefore, this paper adopts tent maps to initialize the artificial fish swarm. The step of the tent map are as follows:

(1) Randomly generate the initial chaotic vector $Z_{0}=\left(Z_{0}^{1},Z_{0}^{2},\ldots Z_{0}^{j}\ldots ,Z_{0}^{D}\right)$, where *D* is the number of parameters to be optimized, which is set to 2 in this paper. The vector $Z_{0}$ should avoid falling into the small cycle (0.2,0.4,0.6,0.8) [17].
(2) Assumed that the maximum iteration number of tent maps is *M*. The expression of the tent map is given by:
  (1) $$Z_{t+1}=\left\{ \begin{array}{l} 2Z_{t},\;\;\;\;\;\;\;\;\;\;\;\;0\leq Z_{t}\leq 0.5\\ 2(1-Z_{t}),\;\;0.5\leq Z_{t}\leq 1 \end{array}\right.$$
  After Bernoulli shift transformation [18], it can be expressed as:
  (2) $$Z_{t+1}=\left(2Z_{t}\right)\mathrm{mod}l.$$
  where t=0,1,…,M, $Z_{t}\in \left(0,1\right)$.
(3) After the iteration is performed by Equation (2), $Z_{t+1}$ is obtained.
(4) If the vector $Z_{t}$ falls into the small cycle {0,0.25,0.5,0.75} or a fixed point during the iteration, the initial value of the iteration is changed according to the equation $Z_{t}=Z_{t-1}+\varepsilon$ [19], where ε is a random number. Otherwise, step (3) continues to be performed.
(5) If the current iteration reaches *M*, the mapping is terminated and the final tent chaotic vector $Z_{M}=\left(Z_{M}^{1},Z_{M}^{2},\ldots Z_{M}^{j}\ldots Z_{M}^{D}\right)$ is obtained. Otherwise, step (3) continues to be performed.

Reverse learning is an intelligent optimization method proposed by Tizhoosh [20]. The main idea is to expand the population by finding the inverse solution of the candidate solution. According to probabilistic analysis, for each randomly generated candidate solution, the corresponding reverse solution is approximately 50% more likely than the candidate solution to approach the global optimal solution [21]. So reverse learning can expand the search range of the population and increase the diversity of the population.

Thus, tent maps and reverse learning are combined in this paper to initialize the population of AFSA. The main idea is to first use the tent chaotic sequence to generate initial candidate solutions $X_{i}$, and then obtain the reverse solution $Y_{i}$ of each candidate solution according to the reverse learning strategy. After the fitness values of all solutions are calculated, the individuals with higher fitness values can be selected as the initial population. The reverse solution in reverse learning is calculated by Equation (3).

(3) $$Y_{i}^{j}=K\left(X_{\min}^{j}-X_{\max}^{j}\right)-X_{i}^{j}$$

where $X_{i}^{j}$ is the jth dimension vector of the ith initial solution of the chaotic map. $Y_{i}^{j}$ represents the jth dimension vector of the reverse solution obtained from the ith initial solution of the chaotic map. $X_{\max}^{j},X_{\min}^{j}$ is the maximum and minimum values of the jth dimension vector, respectively. K is a random number within [0, 1]. Based on tent maps and reverse learning, the step to initialize the population is as follows:

(1) Firstly, randomly generate the initial chaotic vector $Z_{0}=\left(Z_{0}^{1},Z_{0}^{2},\ldots Z_{0}^{j}\ldots ,Z_{0}^{D}\right)$. According to the step of the tent map, when the iteration reaches *M*, the final tent chaotic vector $Z_{M}=\left(Z_{M}^{1},Z_{M}^{2},\ldots Z_{M}^{j}\ldots Z_{M}^{D}\right)$ is obtained.
(2) The initial candidate solutions $X_{i}=\left(X_{i}^{1},X_{i}^{2},\ldots X_{i}^{j}\ldots ,X_{i}^{D}\right),\left(i=1,2,\ldots ,S\right)$ can be obtained by the equation $X_{i}^{j}=X_{\min}^{j}+Z_{M}^{j}\times \left(X_{\max}^{j}-X_{\min}^{j}\right)$.
(3) According to the Equation (3), the reverse solution $Y_{i}=\left(Y_{i}^{1},Y_{i}^{2},\ldots Y_{i}^{j}\ldots ,Y_{i}^{D}\right)$ of $X_{i}=\left(X_{i}^{1},X_{i}^{2},\ldots X_{i}^{j}\ldots ,X_{i}^{D}\right)$ can be obtained, where i=1,2,…,S.
(4) Calculate the fitness values of $X_{i}$ and $Y_{i}$, and sort the fitness values from high to low. The first *S* individuals with higher fitness are selected as the initial population of AFSA.

### 2.2. Adaptive Vision and Step

AFSA uses a fixed vision and step when optimizing parameters, which is not reasonable. The vision affects the search range of the artificial fish, and the step affects the iteration speed and searching accuracy of the algorithm. If the selection of the vision and step is adaptive, it can greatly improve the performance of AFSA.

In this paper, the Lorentz function of the Cauchy distribution is introduced in Equation (4) so as to adaptively adjust the vision, and the normal distribution function is introduced in Equation (5) to adaptively adjust the step [22].

(4) $$f\left(x;x_{0},\gamma ,I\right)=I\left[\frac{\gamma^{2}}{{\left(x-x_{0}\right)}^{2}+\gamma^{2}}\right]$$

(5) $$g\left(x\right)=e^{-\pi x^{2}}$$

**Figure 1:**
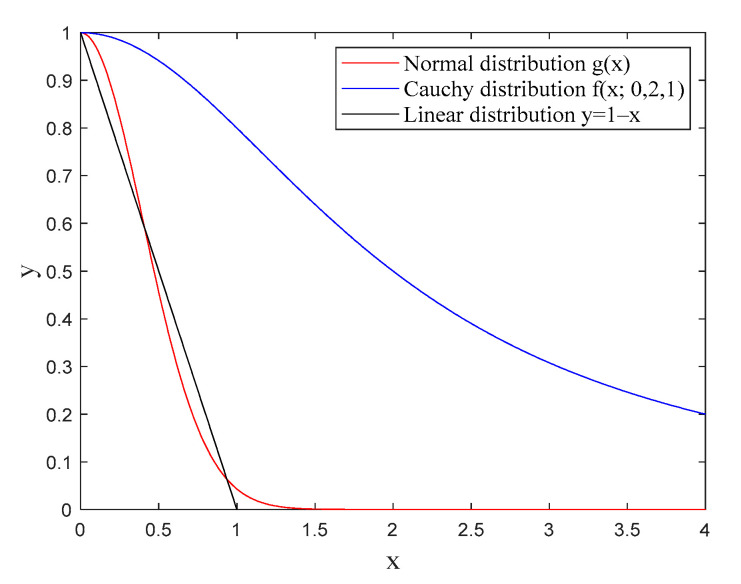
Comparison of Cauchy and normal distribution with the linear distribution.

Therefore, the adaptive adjustment equations of the vision and step are given by:

(6) $$\left\{ \begin{array}{l} Visual_{t+1}=Visual_{t}\times f\left(\frac{4\cdot t}{T_{\max}};0,2,1\right)+Visual_{\min}\\ Step_{t+1}=Step_{t}\times g\left(\frac{t}{T_{\max}}\right)+Step_{\min} \end{array}\right.$$

where, $Visual_{\min}$ is the minimum value of the vision. $Step_{\min}$ is the minimum value of the searching step. t and $T_{\max}$ are the current iteration number and the maximum iteration number, respectively. The function curves of the adjusting vision and step compared with the linear distribution curve are shown in Figure 1.

It can be seen from Figure 1 that the normal distribution has a slower decline rate than the linear function in the initial stage of searching, so the searching step of IAFSA is larger, which increases the searching speed. In the later stage of searching, the function value of the normal distribution is always smaller than that of the Cauchy distribution, so the searching step of IAFSA is smaller, which can achieve accurate search. Because the decline rate of the Cauchy distribution is slower than that of the normal distribution, the vision of IAFSA is changed less. It illustrates that IAFSA is not easy to fall into precocity. This improved method makes the algorithm have a faster searching speed and a stronger global searching capability at the beginning, and a higher searching accuracy in the later period.

### 2.3. Adaptive t Distribution Mutation

In 1908, W.S. Gosset proposed the *t* distribution which regards the degree of freedom *n* as a variable [23]. The probability density function is given by:

(7) $$p_{t}\left(x\right)=\frac{\Gamma \left(\frac{n+1}{2}\right)}{\sqrt{n\pi }\cdot \Gamma \left(n/2\right)}\cdot {\left(1+\frac{x^{2}}{n}\right)}^{-\frac{n+1}{2}},-\infty <x<+\infty$$

where *n* is the degree of freedom and Γ(⋅) is the gamma function. When n=1 in Equation (7), the distribution curve is Cauchy distribution C(0,1). When n>30, the curve begins to coincide with the normal distribution. When n→∞, the *t* distribution is similar to the Gaussian distribution N(0,1). Therefore, Cauchy distribution C(0,1) and Gaussian distribution N(0,1) are special cases of the *t* distribution.

The Cauchy distribution can generate random numbers farther from the origin with a higher probability, which can make the algorithm have a better global development performance [24]. The probability of random numbers generated by Gaussian distribution near the origin is relatively large, so the Gaussian mutation has strong local development performance [25]. In this paper, adaptive *t* distribution is used to mutate the population. The mutation process is as follows:

Assumed that η is the control factor which is used to control the degree of mutation, and the calculation method is shown in Equation (8).

(8) $$\eta =1-\frac{i}{T_{\max}-1}$$

where$\;i=0,1,2,\ldots ,T_{\max}-1$. $T_{\max}$ is the maximum number of iterations. η is an arithmetic sequence that gradually decreases from 1 to 0. When searching for the first time, η=1 and it indicates that the coefficient of mutation is fully functional. In the last search, η=0 and it indicates that the coefficient of mutation does not work.

Therefore, the position $X_{i}$ of artificial fish to perform the adaptive *t* distribution mutation is calculated in Equation (9).

(9) $$X_{i}^{*}=X_{i}+\eta \cdot t\left(j\right)\cdot X_{i}$$

where $\;j=1,2,\ldots ,T_{\max}$. $T_{\max}$ is the maximum number of iterations. $X_{i}^{*}$ is the position of the ith artificial fish after mutation, and $X_{i}$ is the position of the ith artificial fish. η is the control coefficient, and t(j) is the random number generated by the *t* distribution with the number of iterations *j* as the degree of freedom.

**Figure 2:**
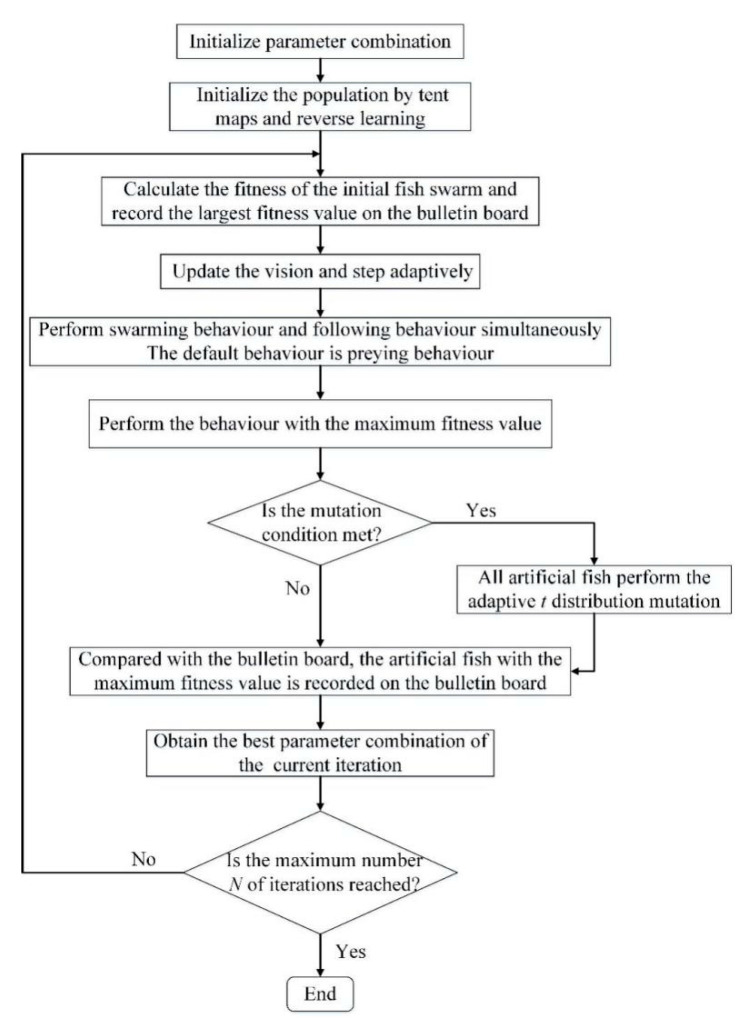
The flow chart of IAFSA.

Therefore, in the initial stage of searching, the number of iterations *j* is small, and the *t* distribution is similar to the Cauchy mutation distribution, so the global development performance of the algorithm in the early stage is high. In the final stage of searching, the number of iterations *j* is large. Additionally, the *t* distribution is similar to the Gaussian mutation distribution, so the local development performance of the algorithm in the late stage is high. The flow chart of IAFSA is shown in Figure 2.

### 2.4. The LSSVM Optimized by IAFSA

**Figure 3:**
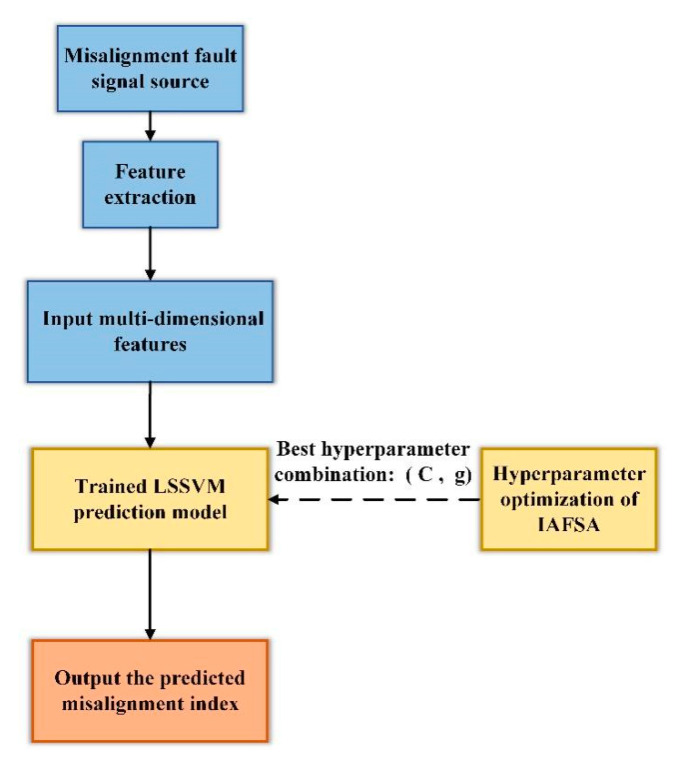
The flow chart of the LSSVM prediction model optimized by IAFSA.

The prediction accuracy is one of the most important indicators of the prediction model. Because the LSSVM model contains two hyperparameters which are the penalty factor C and kernel parameter g. If the selection of these two parameters is inappropriate, it will cause a decrease in prediction accuracy and an increase in the false alarm rate. Manual adjustment of parameters does not have a certain theoretical basis, which consumes a lot of time. Therefore, the improved AFSA (IAFSA) is used to optimize the hyperparameters of LSSVM, and the root mean square error (RMSE) between the predicted value and the true value is used as the objective function to obtain the optimal hyperparameter combination of LSSVM. Based on the multi-dimensional features of misalignment signal, the LSSVM optimized by IAFSA can predict the future development trend of the misalignment index. The flow chart of the LSSVM model optimized by IAFSA is shown in Figure 3.

## 3. Data Collection and Feature Selection of Misalignment Fault

### 3.1. Data Collection of Misalignment Fault

**Figure 4:**
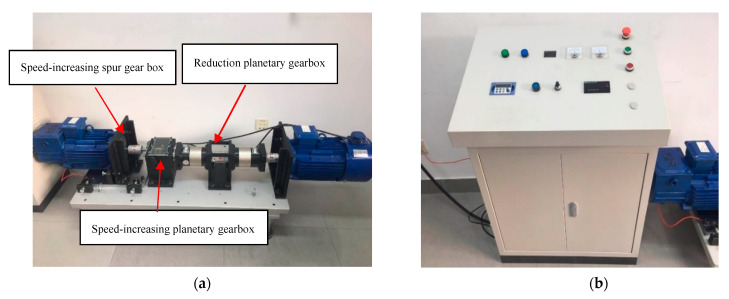
The test bench of misalignment fault for wind turbines. (**a**) experiment platform; (**b**) control cabinet.

The prediction data of misalignment fault is collected from the test bench which is mainly composed of the electric motor (active motor), transmission system, generator (passive motor), and control cabinet, as is shown in Figure 4. The model of active motor is YP2-112M-6, which is the variable-frequency variable-speed three-phase asynchronous motor. The rated power is 2.2 kW, the rated voltage is 380 V, the rated current is 5.6 A, the maximum speed is 940 r/min, and the number of poles is 6. The rotation speed of the motor is first reduced by the planetary gearbox with a transmission ratio of 1:50 in order to correspond to the rotation speed of the wind wheel. To simulates the operation of wind turbines, the rotation speed is then increased by the planetary gearbox with a transmission ratio of 40:1 and the spur gear box with a transmission ratio of 1.5:1. The generator is transformed from YZR112M-6-1.5 kW, the rated power is 1.5 kW, the maximum speed is 1000 r/min, and the number of poles is 6. The generator, motor, gearbox, and other components are fixed on the base table, which is convenient for setting misalignment fault. The speed of the motor can be set on the control cabinet. Besides, the control cabinet is equipped with a start–stop button and a running indicator to ensure the safe operation of the test bench.

**Figure 5:**
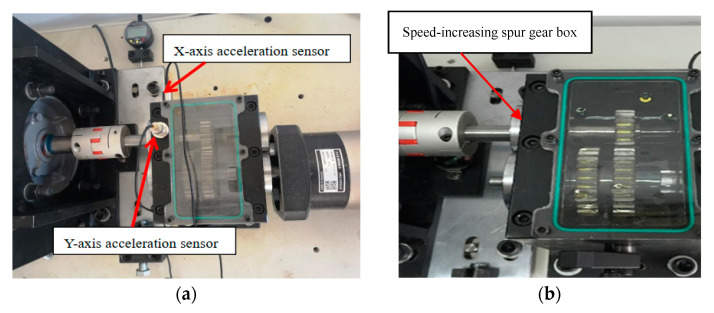
The position of the acceleration sensor. (**a**) the vibration signal collection; (**b**) the spur gear box.

**Figure 6:**
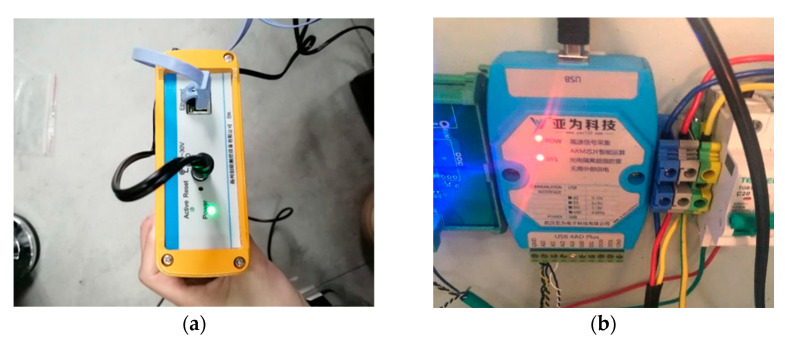
The signal acquisition device of test bench. (**a**) the DFT5100 dynamic data collector; (**b**) the signal acquisition card USB 4AD Plus.

The experimental platform realizes the simulation of misalignment fault by adjusting the position of the generator. Both the angle sensor and the displacement sensor are located on the base table. In addition, two acceleration sensors are placed on the end of the spur gear box near the coupling in the *X*-axis and *Y*-axis directions to collect radial vibration, as is shown in Figure 5. During the experiment, the DFT5100 dynamic data collector in Figure 6a from the two acceleration sensors is adopted to collect vibration signals of misalignment fault [26]. At the same time, the stator current signal is transmitted to the USB signal collection and recording platform by the signal acquisition card USB 4AD Plus which is shown in Figure 6b to realize the collection and display of stator current signals [26].

**Figure 7:**
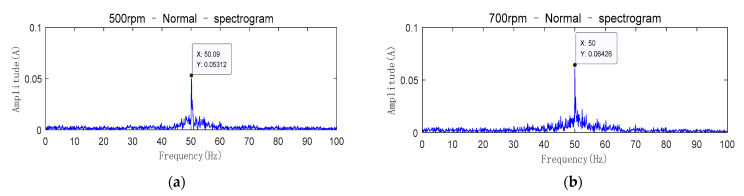
Comparison graph of variable speed constant frequency (**a**) The generator speed is 600 rpm; (**b**) The generator speed is 840 rpm.

The double-fed wind turbine can realize the operation state of variable speed constant frequency. In order to validate whether the test bench can show the response of the real wind turbine, changing the motor speed and observing whether the frequency of stator current remains the same to prove the effectiveness of the wind turbine simulation test bench. The sampling frequency of stator current is 2 kHz. When the motor speed is 500 rpm, the rotor speed of the generator is 600rpm due to the 1.2 times transmission ratio. Figure 7a shows the frequency domain waveform of the stator current at this time. When the motor speed is changed to 700 rpm, the rotor speed of the generator is 840 rpm due to the 1.2 times transmission ratio. Figure 7b shows the frequency domain waveform of the stator current at this time.

In Figure 7, only the frequency component of 50 Hz is the most prominent and the frequency of stator current is the same at different speeds. Therefore, the test bench can successfully realize the operation state of variable speed constant frequency under the control of a closed-loop.

**Figure 8:**
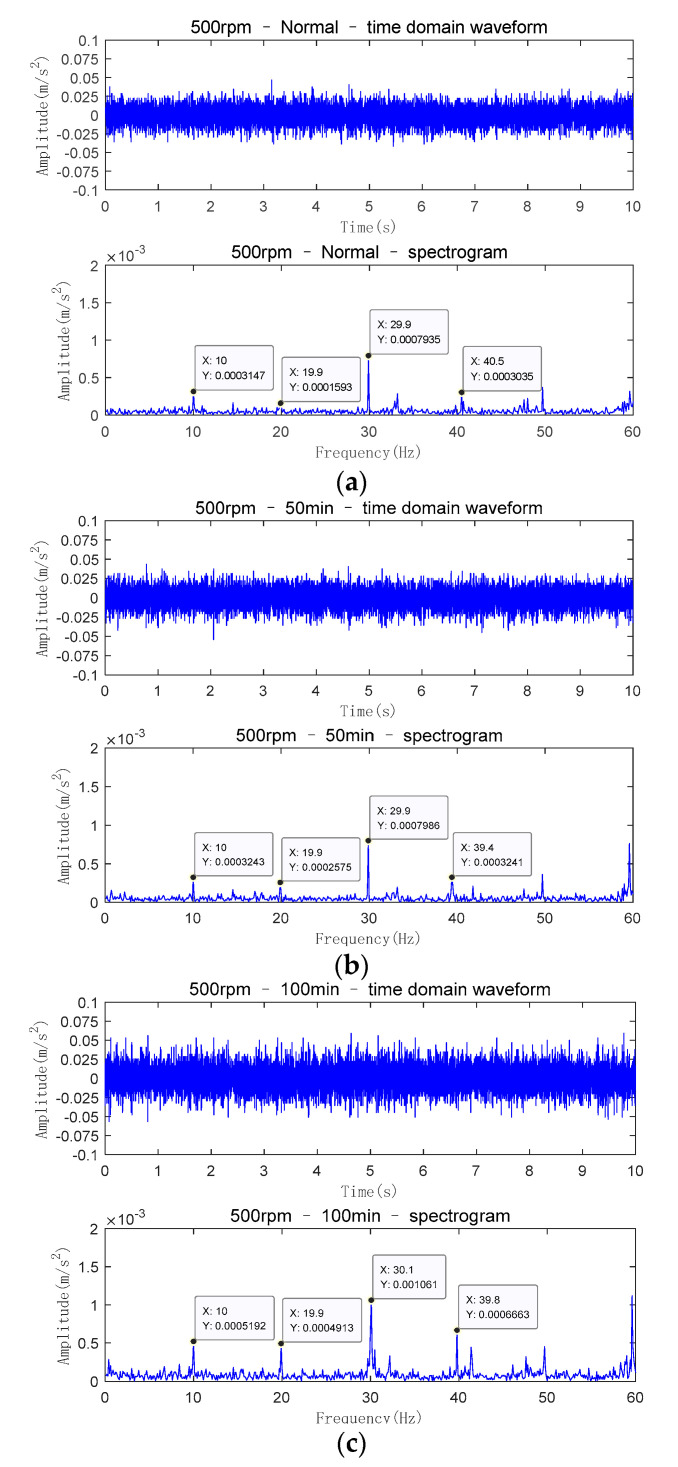
Time domain waveform and spectrogram of vibration signals. (**a**) normal operation condition; (**b**)after running 50 min; (**c**) after running 100 min.

**Figure 9:**
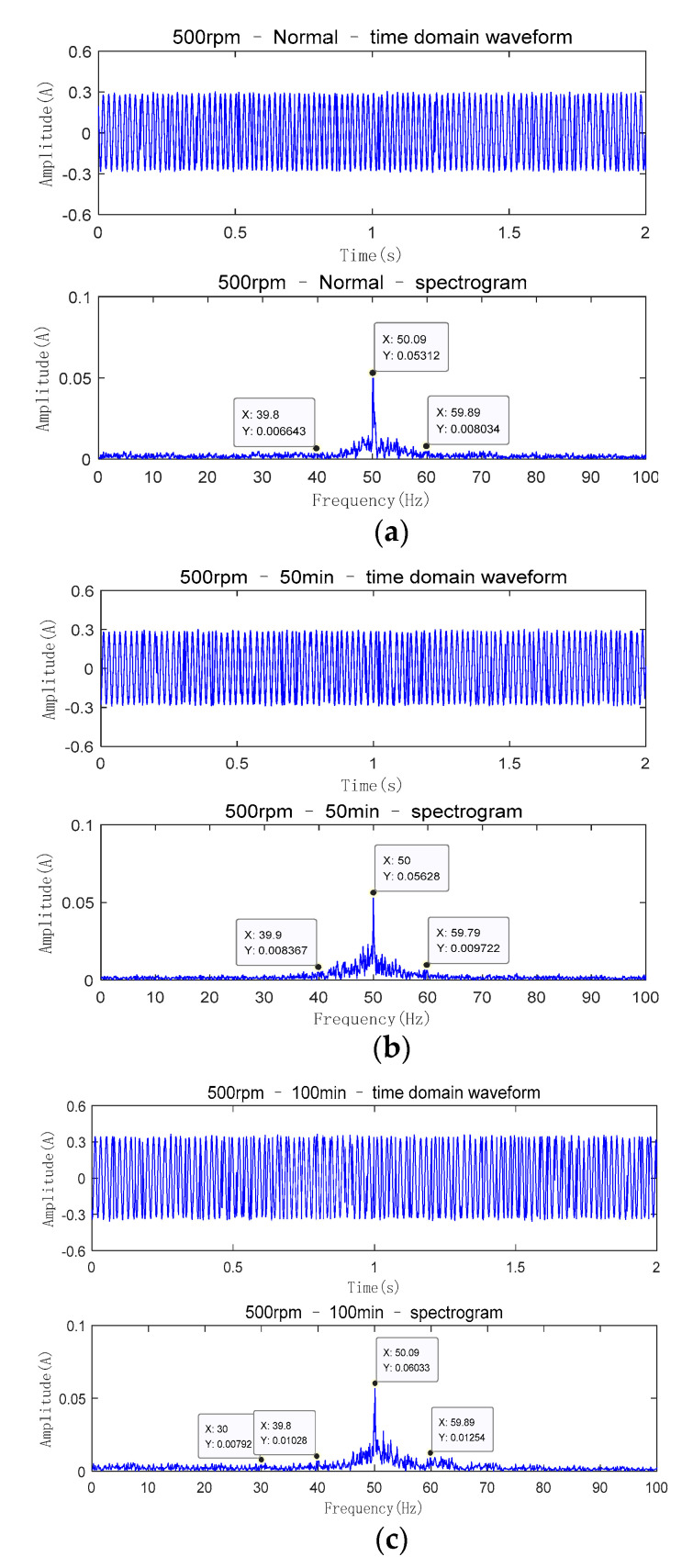
Time domain waveform and spectrogram of stator current signals. (**a**) normal operation condition; (**b**)after running 50 min; (**c**) after running 100 min.

In the experiment, for the collection of signals, the rotation speed of the motor is set to 500 rpm and the sampling interval is 5 min. The sampling time of the vibration signal is 10 s and the sampling frequency of acceleration sensor is 1 kHz. The sampling time of the current signal is 2 s and the sampling frequency of the current signal is 2 kHz. During the experiment, a slight misalignment fault occurred between the output shaft of the gearbox and the rotor shaft of the generator by adjusting the position of the generator. At the same time, 60 samples of vibration and current signals under misalignment fault are collected. The time-domain waveform and spectrogram of vibration signals and current signals collected by the experimental platform at different running times are shown in Figure 8 and Figure 9, respectively.

As the motor speed is 500 rpm, the speed transmitted to the generator rotor shaft is 600 rpm after 1.2 times the total transmission ratio. According to the mathematical conversion between speed *n* and frequency $f_{r}$: $f_{r}=n/60$, it can be concluded that the rotating frequency of the generator rotor shaft is 10 Hz. Theoretically, when the misalignment fault occurs, there will be multiple frequency multipliers such as fundamental frequency 10 Hz, the second harmonic 20 Hz and the third harmonic 30 Hz in the spectrum [27].

It can be seen from Figure 8 that the characteristic frequency obtained from the signal of the experimental platform is basically consistent with the theoretical frequency. Because in the process of actual misalignment fault, comprehensive misalignment fault is a common form, and with the increase in fault degree, the amplitudes of the first and second harmonic in the spectrum will increase [27]. In Figure 8a–c, the amplitude of the time domain waveform and the amplitude of the first and second harmonic in the spectrum gradually increase, but the increase is not very obvious. It shows that the fault characteristics are not obvious and it is a latent fault when the misalignment fault occurs in the early stage. Therefore, it is necessary to predict the fault trend of misalignment fault and establish a reasonable fault early warning threshold, so as to take timely remedial measures before the fault is serious and realize fault early warning.

Because the misalignment fault in the test bench occurs at the coupling connection between the speed-increasing gearbox output shaft and the generator rotor shaft, it belongs to the coupling misalignment fault. According to the Reference [28], this misalignment fault will cause mechanical disturbance. Any mechanical disturbance of the generator rotor will cause the change of the air gap magnetic flux waveform, which will cause the change of the stator current. The details can be given by the following formula:

(10) $$f_{e}=f_{l}\pm mf_{r}$$

where $f_{l}$ is the supply frequency, which is 50 Hz in this paper. $f_{r}$ is the rotation frequency of the rotor and *m* = 1, 2, 3...... harmonic number. $f_{e}$ is the current harmonic component due to air gap disturbance. It can be seen from the Formula (10) that when a misalignment fault occurs in the transmission system, the misalignment fault information will be mapped to the stator current. Since the motor speed is set to 500 rpm, the speed transmitted to the generator rotor shaft is 600 rpm. From the mathematical conversion between speed and frequency, the rotation frequency can be obtained as 10 Hz. Theoretically, when a misalignment fault occurs, there will be 50 − 2 × 10 = 30 Hz, 50 − 10 = 40 Hz, 50 + 10 = 60 Hz and 50 + 2 × 10 = 70 Hz and other fault frequencies, in addition to the power frequency 50 Hz [29].

It can be seen from Figure 9 that the characteristic frequency obtained from the signal of the experimental platform is basically consistent with the theoretical frequency. The amplitudes of power frequency and fault frequency increase with the increase in operation time. It can be seen from Figure 9a–c that the increase amplitude is small at the beginning, so it is not easy to find fault features, and the amplitude of fault frequency is less than that of power frequency, so it has randomness to judge whether there is early fault through the current spectrum. Therefore, it is necessary to extract fault features and use a prediction model to predict misalignment fault, so as to achieve a more accurate early fault warning.

### 3.2. Input and Output Features of Prediction Model for Misalignment Fault

The LSSVM optimized by IAFSA is used as the prediction model of misalignment fault for wind turbines in this paper. The input of the prediction model is the normalized fault feature vector which is constructed by time domain features, frequency domain features, and time-frequency domain features. The input features of vibration and current signals for fault prediction are listed in Table 2 and Table 3, respectively. The theoretical basis of the index selection is in reference [30] and the detailed contents of Table 2 and Table 3 is consistent with reference [30]. The method adopted in reference [30] is mainly that the combined forecasting model improves the prediction accuracy of the simulation misalignment fault, and the optimization algorithm is not improved. However, the method proposed in this paper is to use an improved artificial intelligence algorithm to optimize the LSSVM model to improve the prediction accuracy of misalignment faults, and to verify it based on the data collected by the misalignment test bench.

The kurtosis index is a dimensionless index, which is given by:

(11) $$K_{f}={\frac{1}{N}\sum_{i=1}^{N}x_{i}^{4}/\left(\sqrt{\frac{1}{N}\sum_{i=1}^{N}x_{i}^{2}}\right)}^{4}$$

where $x_{i}\left(i=1,2,\ldots ,N\right)$ stands for each signal sample, and *N* represents the number of data points in the signal sample.

When the equipment is in normal operation, the amplitude distribution of signals is approximate to the normal distribution. When the equipment happens early faults, the amplitude of signals will gradually increase and the probability density distribution will become steeper; thereby, the amplitude distribution will appear skewed, gradually deviating from the normal distribution. Meanwhile, the value of the kurtosis index will increase as the deviation or steepness increases. The experiment shows that the value of the kurtosis index shows an obvious tendency in the research of equipment fault detection and prediction. When the equipment is in normal operation, the kurtosis index is about three. When the equipment has early faults, its value will increase significantly [31]. This phenomenon demonstrates that the kurtosis index has high sensitivity and regularity to early faults and impact signals. Therefore, the kurtosis index is selected as the output of the prediction model for misalignment fault in this paper.

## 4. Misalignment Fault Prediction Based on IAFSA

The vibration signals and current signals obtained from the experimental platform are taken as the feature signals. The first 45 samples of the collected 60 fault samples are used as the training set of LSSVM, and the last 15 samples are used as the testing set of LSSVM. Six optimization methods are adopted to optimize the parameters of LSSVM, which are the Improved Artificial Fish Swarm Algorithm (IAFSA), Artificial Fish Swarm Algorithm (AFSA), Particle Swarm Optimization (PSO), Quantum Genetic Algorithm (QGA), Genetic Algorithm (GA) and Grid Search method (GridSearch). Among them, the initial parameters of IAFSA and AFSA are set as follows: The crowding factor is 0.618 and the maximum number of attempts is 10. The step is 0.5 and the vision is 0.66. The iteration number of tent maps in IAFSA is 10. The initial parameters of PSO are set as follows: The local search ability $c_{1}$ = 1.5 and the global search ability $c_{2}$ = 1.7 [32]. The initial parameter setting of QGA is set as follows: The coding length of the quantum chromosome is 20. The initialization parameters of GA are set as follows: The crossover probability is 0.9 and the mutation probability is 0.1 [33]. For all algorithms, the population is set as 20 and the maximum number of cycles *N* is set as 200. The search range of the penalty parameter of LSSVM is [0.01, 100], and the search range of the radial basis function kernel is [0.01, 1000].

### 4.1. Prediction Results of Misalignment Fault Based on Vibration Signals

**Figure 10:**
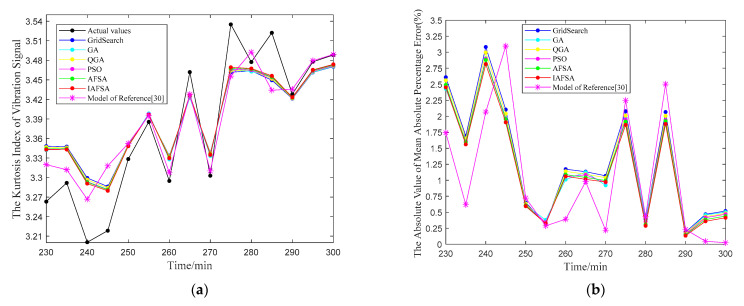
The vibration signal prediction results of LSSVM optimized by six algorithms.

For the vibration signals acquired from the test bench, the predicted results of LSSVM optimized by six algorithms and the combination prediction of Reference [30] are shown in Figure 10.

In Figure 10a, the predicted value of IAFSA is relatively closer to the original data, and Mean Absolute Percentage Error (MAPE) is the smallest at most of the predicted points in Figure 10b. The prediction index obtained from the above six optimization methods and the combination prediction of Reference [30] are shown in Table 4.

It can be concluded from Table 4 that:

(1) In the test set, compared with other algorithms, the RMSE and MAPE indexes of IAFSA_LSSVM are the smallest, which indicates that the prediction error of LSSVM optimized by IAFSA is the smallest. In addition, the *R^2^* index of IAFSA_LSSVM is the largest among all the algorithms, which means that the changing trend predicted by IAFSA_LSSVM is the most consistent with that of real data. Comprehensive comparison of the above three indicators, IAFSA_ LSSVM has the best prediction effect in the testing set, followed by the combination prediction of Reference [30], AFSA, PSO, GA, QGA and GridSearch.
(2) In the training set, the RMSE and MAPE indexes of IAFSA_LSSVM are the smallest, and the *R^2^* index is the largest. Therefore, the IAFSA_LSSVM has the highest prediction accuracy, followed by the combination prediction of Reference [30], AFSA, QGA, GridSearch, PSO and GA.

Thus, for the prediction of the vibration signals of misalignment fault, the IAFSA_LSSVM prediction model has the best prediction accuracy in the training set and testing set. It shows that the IAFSA algorithm has certain advantages.

### 4.2. Prediction Results of Misalignment Fault Based on Current Signals

**Figure 11:**
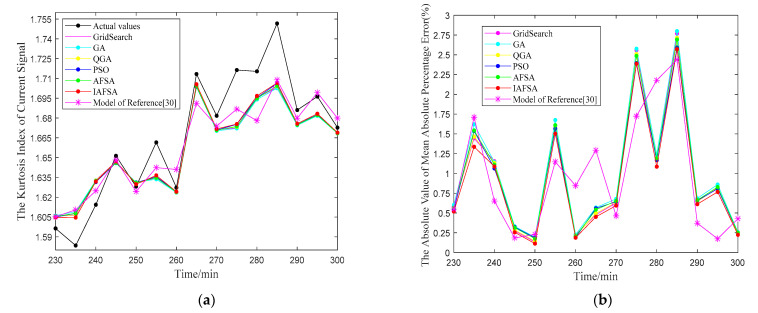
The current signal prediction results of LSSVM optimized by six algorithms.

For the current signals collected from the test bench, the predicted results of LSSVM optimized by six algorithms and the combination prediction of Reference [30] are shown in Figure 11.

In Figure 11a, the predicted point of the LSSVM optimized by IAFSA is closer to the original actual value. In Figure 11b, the MAPE values of the LSSVM optimized by IAFSA are all below 3%, and it is the smallest in most data points. The prediction index obtained from the above six optimization methods and the combination prediction of Reference [30] are shown in Table 5.

It can be seen from Table 5 that:

(1) In the test set, IAFSA_ LSSVM has the smallest RMSE and MAPE, and it has the largest *R^2^*, which indicates that the prediction error of IAFSA_ LSSVM is the smallest, and the fitting degree of IAFSA_ LSSVM between the predicted value and the real value is the best, followed by the combination prediction of Reference [30], PSO, QGA, AFSA, GridSearch and GA.
(2) In the training set, Comprehensive comparison of RMSE, MAPE and *R^2^*, IAFSA_ LSSVM has the highest prediction accuracy, followed by GridSearch, QGA, the combination prediction of Reference [30], AFSA, GA and PSO.

Therefore, when predicting current signals of misalignment fault, the IAFSA_ LSSVM prediction model not only has the best prediction effect in the training set, but also has the highest prediction accuracy in the testing set.

In conclusion, according to the prediction results in Table 4 and Table 5, the LSSVM optimized by IAFSA can more accurately predict the development trend of signals, which contributes to achieving accurate fault prediction.

### 4.3. Realization of Misalignment Fault Warning

Misalignment fault is a kind of latent fault, and its early fault characteristics are not very obvious. Therefore, it is necessary to set a warning line for misalignment fault, which contributes to making a reasonable remedy plan before the fault deteriorates. Because the kurtosis index has a high sensitivity to the early fault and impact signal, it is adopted to set the warning line of misalignment fault. When the equipment is in a normal operation state, the characteristics of signal distribution are close to the normal distribution [34]. Therefore, the 3σ principle of the normal distribution can be taken as the relative standard to obtain the fault warning line.

**Figure 12:**
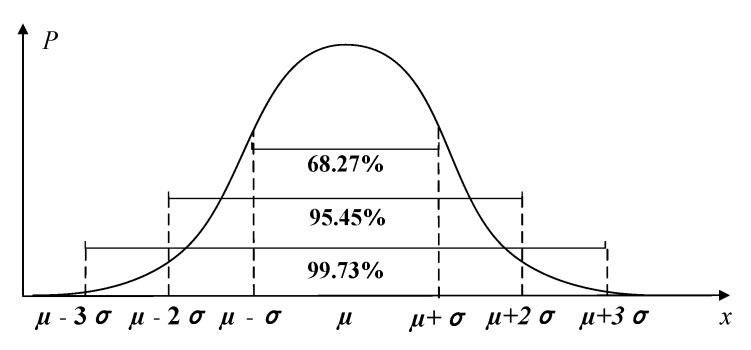
The probability distribution of 3σ principle.

Assuming that the random variable X follows the normal distribution. The average value of X is μ and the standard deviation is σ. From the 3σ principle, the probability of data falls within the range (μ−σ,μ+σ) is P=68.27%. The probability of data falls within the range (μ−2σ,μ+2σ) is P=95.45%. The probability of data falls within the range (μ−3σ,μ+3σ) is P=99.73% [35], as shown in Figure 12. According to the characteristics of the normal distribution, when the equipment is in a normal state, the probability of the kurtosis index falls within the range (μ−3σ,μ+3σ) is P=99.73%. When the fault occurs, the distribution of fault signals gradually deviates from the normal distribution. Meanwhile, the value of the kurtosis index obtained from signals will exceed the range (μ−3σ,μ+3σ), belonging to abnormal data. Thus, μ+3σ and μ−3σ calculated from the kurtosis index are taken as the upper and lower limits of misalignment fault warning. If the kurtosis index obtained from collected signals is within (μ−3σ,μ+3σ), it represents that the equipment is still in normal operation. If the kurtosis index is outside the range (μ−3σ,μ+3σ), it demonstrates that the equipment is in an abnormal state, which achieves the misalignment fault warning.

According to the 3σ principle, the warning line of kurtosis index can be set as follows:

(12) $$K_{up}=\mu_{K}+3\cdot \sigma_{K}$$

where $\mu_{K}$ is the average value of the kurtosis index under the normal operation of equipment. $\sigma_{K}$ is the standard deviation of the kurtosis index. Therefore, when the kurtosis index exceeds the warning value $K_{up}$, it indicates that the fault degree exceeds the acceptable range for the normal operation of equipment.

This paper adopts 20 historical samples of the vibration and current signals of the wind turbine experiment platform during normal operating conditions. The average value and standard deviation of the kurtosis index of vibration and current signals during normal operation are listed in Table 6.

**Figure 13:**
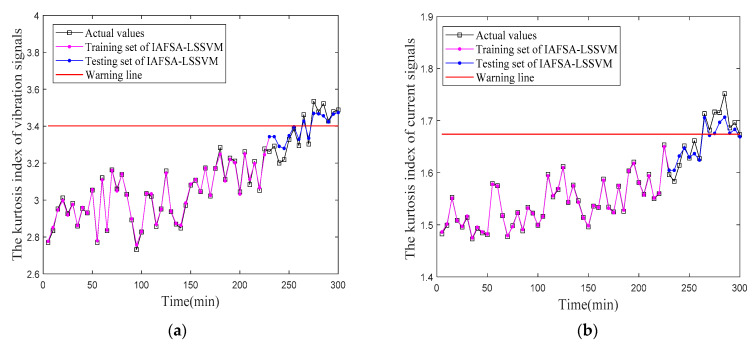
Fault warning lines. (**a**) vibration signals; (**b**) current signals.

According to Table 6, the warning lines of vibration and current signals can be obtained, as shown in Figure 13 (red).

Figure 13 shows the experimental process in which the misalignment faults continue to increase. It can be seen from Figure 13 that the predicted kurtosis index obtained from the training set and testing set of LSSVM optimized by IAFSA is basically close to the actual values of vibration and current signals. In Figure 13, the kurtosis index of vibration and current signals increases with the rise of the operation time of the test bench, which indicates that the fault degree of misalignment also grows up with the increase in running time. Before 265 min, although the kurtosis index of vibration and current signals has a certain increase trend, it does not exceed the warning line. This is mainly because the fault degree of misalignment is minor at the beginning of the test bench, which is not sufficient for having a large impact on the normal operation. Therefore, the kurtosis index does not exceed the warning line. At 265 min, the kurtosis index of vibration and current signals has exceeded the warning line, and most of the kurtosis index values are higher than the warning line after 265 min. It can be known from the 3σ principle that the kurtosis index of vibration and current signals at 265 min no longer belongs to the fluctuation range of the kurtosis index during the normal operation of equipment, but belongs to abnormal data. This indicates that the fault degree of misalignment has exceeded the acceptable range of normal operation. Therefore, when the test bench runs to 265 min, it should be shut down in time to prevent the damage of equipment due to the continued deterioration of misalignment faults, so as to achieve the early warning.

## 5. Conclusions

In this paper, the AFSA is improved and it is used to optimize the parameters in LSSVM to predict the kurtosis index of vibration signals and stator current signals for wind turbines misalignment fault. The main conclusions obtained in the research are as follows:

1. After analyzing the collected misalignment fault vibration and current signals in the time domain and frequency domain, it is proved that the early fault signs of misalignment are not very obvious, and the misalignment fault is a slowly changing latent fault.
2. The AFSA is improved. The IAFSA can find the optimal hyperparameter solution of the LSSVM model, and its prediction accuracy is the highest compared with other optimization algorithms.
3. According to the 3σ principle of the normal distribution, the early warning line of vibration and current signals is set. The experimental results prove that the proposed prediction model achieves a relatively accurate prediction and early warning for misalignment fault of wind turbines.

Due to the limitations of the current laboratory misalignment fault data, we can only test and verify the misalignment fault data obtained from the wind turbine misalignment simulation test bench. If the actual operating state data of the wind turbine is obtained in the future, it can be used as one of the inputs of the prediction model for fault prediction research, thereby improving the adaptability of the prediction model. In future research, the IAFSA-LSSVM model will be considered for use in other fault prediction fields.

## Figures and Tables

**Table 1:** The commonly used prediction algorithms.

Method	Scope	Advantage	Disadvantage
ARIMA	Small sample Linear prediction	Less samples required Simple model	Not suitable for complex nonlinear forecasts
RF	Small or medium sample Non-linear prediction	Fast training Automatic feature selection	Poor robustness Many optimization parameters
KF	Small or medium sample Linear prediction	Dynamic modeling Good robustness	Not suitable for complex nonlinear forecasts
LSSVM	Small or medium sample Non-linear prediction	High accuracy predictions based on few samples	Model parameters affects prediction accuracy
LSTM	Large sample Complex nonlinear prediction	Strong nonlinear ability Processing large data sets	Model parameters affects prediction accuracy Many optimization parameters

**Table 2:** The feature vector indexes of vibration signals.

Feature Vector	Category	Indexes
Vibration signals (9 dimensions)	Time domain	Root mean square, kurtosis, kurtosis index
	Frequency domain	Mean square frequency, center of gravity frequency, frequency variance
	Time-frequency domain	The first three energy entropies of the IMF components obtained by the Improved Empirical Mode Decomposition (IEMD)

**Table 3:** The feature vector indexes of current signals.

Feature Vector	Category	Indexes
Current signals (11 dimensions)	Time domain	Root mean square, kurtosis, kurtosis index
	Frequency domain	Mean square frequency, centre of gravity frequency, frequency variance
	Time-frequency domain	The five sample entropies obtained by the four-layer decomposition of the Dual-tree Complex Wavelet Transform (DTCWT)

**Table 4:** The prediction index of vibration signals.

Method	Data Set	RMSE	MAPE (%)	*R^2^*
IAFSA_LSSVM	Training set	0.0122	0.2966	0.9927
	Test set	0.0477	1.1753	0.8103
AFSA_LSSVM	Training set	0.0127	0.3069	0.9922
	Test set	0.0488	1.2061	0.8012
PSO_LSSVM	Training set	0.0146	0.3632	0.9896
	Test set	0.0491	1.2182	0.7991
QGA_LSSVM	Training set	0.0132	0.3219	0.9915
	Test set	0.0508	1.2609	0.7845
GA_LSSVM	Training set	0.0177	0.4602	0.9848
	Test set	0.0492	1.2274	0.7985
GridSearch_LSSVM	Training set	0.0139	0.3400	0.9905
	Test set	0.0523	1.3019	0.7718
Combination prediction of Reference [30]	Training set	0.0124	0.3016	0.9923
	Test set	0.0479	1.1891	0.8089

RMSE: Root Mean Square Error; MAPE: Mean Absolute Percentage Error; *R^2^*: coefficient of determination, also known as the goodness of fit, is the ratio of the sum of squared regressions to the sum of squared sums, which can represent the fitting degree of the regression model to data set [30].

**Table 5:** The prediction index of current signals.

Method	Data Set	RMSE	MAPE (%)	*R^2^*
IAFSA_LSSVM	Training set	0.0018	0.1003	0.9982
	Test set	0.0200	0.9082	0.8377
AFSA_LSSVM	Training set	0.0027	0.1445	0.9960
	Test set	0.0213	0.9736	0.8163
PSO_LSSVM	Training set	0.0031	0.1601	0.9949
	Test set	0.0206	0.9601	0.8270
QGA_LSSVM	Training set	0.0022	0.1168	0.9975
	Test set	0.0212	0.9696	0.8176
GA_LSSVM	Training set	0.0029	0.1541	0.9954
	Test set	0.0221	1.0256	0.8013
GridSearch_LSSVM	Training set	0.0019	0.1043	0.9981
	Test set	0.0214	0.9757	0.8130
Combination prediction of Reference [30]	Training set	0.0025	0.1384	0.9964
	Test set	0.0204	0.9542	0.8306

**Table 6:** Statistical parameters of kurtosis index under normal operating conditions.

Kurtosis Index	Average	Standard Deviation	Upper Warning Limit
Vibration signals	2.9385	0.1542	3.4011
Current signals	1.4872	0.0622	1.6738
